# (12,17-Dieth­oxy­carbonyl-11,18-dimethyl-2,3:6,7-dibutano­corrphycenato)copper(II)–12,17-dieth­oxy­carbonyl-11,18-dimethyl-2:3,6:7-dibutano­corrphycene (3/97)

**DOI:** 10.1107/S1600536811052214

**Published:** 2011-12-10

**Authors:** Yoshiki Ohgo, Yuki Yokoyama, Daisuke Hashizume, Saburo Neya, Mikio Nakamura

**Affiliations:** a Department of Chemistry, Faculty of Medicine, Toho University, Ota-ku Tokyo 143-8540, Japan; b Department of Physical Chemistry, Graduate School of Pharmaceutical, Sciences, Chuoh-Inohana, Chiba, Chiba 260-8675, Japan; c Advanced Technology Support, Division, RIKEN Advanced Science, Institute, Wako, Saitama 351-0198, Japan

## Abstract

The corrphycene mol­ecule of the title compound, [Cu(C_36_H_36_N_4_O_4_)]_0.034_.0.966C_36_H_38_N_4_O_4_, has an essentially planar macrocyclic framwork with a slightly distorted trapezoidal N_4_ core; the r.m.s. deviation of the peripheral 20 C atoms and four N atoms is 0.054 (3) Å. The surface area within the N_4_-coordinating core (8.358 Å^2^) is significantly smaller than that (8.503 Å^2^) of the corresponding free-base porphyrin. Two intra­molecular N—H⋯N hydrogen bonds are observed. Detailed structure analysis clarified that the co-crystallization of the free-base corrphycene together with a quite minor component (*ca* 3%) of corrphycenato–Cu^II^ occurred in the recrystallization process.

## Related literature

For the first synthesis of free-base corrphycene, see: Sessler *et al.* (1994 ▶). For some related metal corrphycene compounds, see: Sessler *et al.* (2000 ▶). For related porphyrin analogues such as porphycene, N-confused porphyrins, corroles *etc*. see: Chmielewski *et al.* (1994 ▶); Erben *et al.* (2000 ▶); Furuta *et al.* (1994 ▶); Gross *et al.* (2000 ▶). For structures of five-coordinated halide-ligated iron(III) porphyrin, porphycene and corrphycene complexes, see: Ohgo, Neya, Funasaki *et al.* (2001 ▶); Ohgo, Neya, Ikeue *et al.* (2001 ▶); Ohgo *et al.* (2002 ▶). For the synthesis of the starting materials, see: Neya *et al.* (1998 ▶); Hombrecher & Horter (1992 ▶). For the structure of the corresponding porphyrin free-base, see: Lauher & Ibers (1973 ▶).
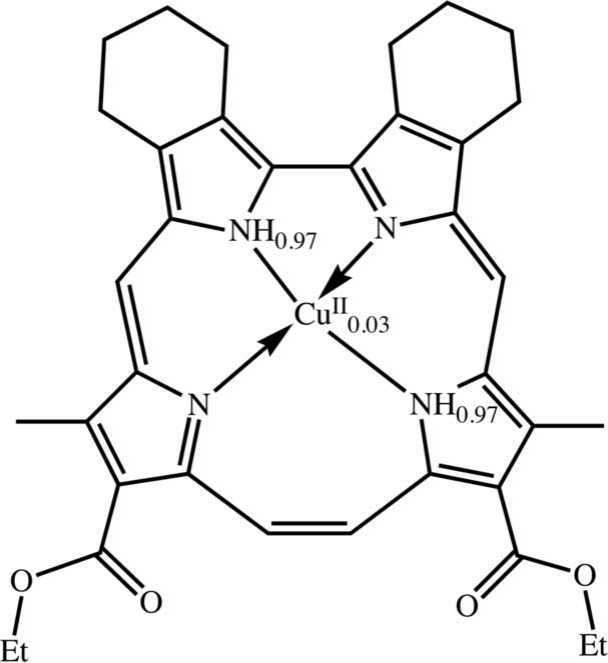


## Experimental

### 

#### Crystal data

[Cu(C_36_H_36_N_4_O_4_)]_0.034_·0.966C_36_H_38_N_4_O_4_
*M* *_r_* = 592.80
Triclinic, 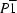

*a* = 8.8759 (5) Å
*b* = 13.2493 (8) Å
*c* = 13.2891 (7) Å
α = 108.496 (2)°
β = 90.708 (2)°
γ = 98.142 (2)°
*V* = 1464.4 (1) Å^3^
*Z* = 2
Mo *K*α radiation
μ = 0.11 mm^−1^
*T* = 296 K
0.31 × 0.25 × 0.10 mm

#### Data collection

Rigaku RAPID diffractometer
Absorption correction: multi-scan (*ABSCOR*; Higashi, 1995 ▶) *T*_min_ = 0.788, *T*_max_ = 0.923
14734 measured reflections
6677 independent reflections
3707 reflections with *I* > 2σ(*I*)
*R*_int_ = 0.044

#### Refinement

*R*[*F* ^2^ > 2σ(*F* ^2^)] = 0.072
*wR*(*F* ^2^) = 0.246
*S* = 1.15
6677 reflections
412 parameters
H-atom parameters constrained
Δρ_max_ = 1.69 e Å^−3^
Δρ_min_ = −0.58 e Å^−3^

### 

Data collection: *CrystalClear* (Rigaku/MSC, 2005 ▶); cell refinement: *HKL-2000* (Otwinowski & Minor, 1997 ▶); data reduction: *HKL-2000*; program(s) used to solve structure: *SIR2004* (Burla *et al.*, 2005 ▶); program(s) used to refine structure: *SHELXL97* (Sheldrick, 2008 ▶); molecular graphics: *ORTEP-3 for Windows* (Farrugia, 1997 ▶); software used to prepare material for publication: *SHELXL97*.

## Supplementary Material

Crystal structure: contains datablock(s) I, global. DOI: 10.1107/S1600536811052214/is5019sup1.cif

Structure factors: contains datablock(s) I. DOI: 10.1107/S1600536811052214/is5019Isup2.hkl

Supplementary material file. DOI: 10.1107/S1600536811052214/is5019Isup4.cdx

Additional supplementary materials:  crystallographic information; 3D view; checkCIF report

## Figures and Tables

**Table 1:** Hydrogen-bond geometry (Å, °)

*D*—H⋯*A*	*D*—H	H⋯*A*	*D*⋯*A*	*D*—H⋯*A*
N3—H3⋯N2	0.85	2.33	2.777 (4)	113
N1—H1⋯N4	0.89	2.11	2.774 (4)	131

**Fig. 1.**
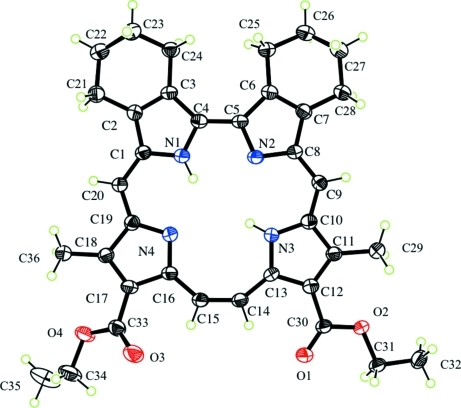
The molecular structure of the title compound with atomic numbering. Displacement ellipsoids are shown at the 50% probability level. Hydrogen atoms are omitted for clarity.

**Fig. 2.**
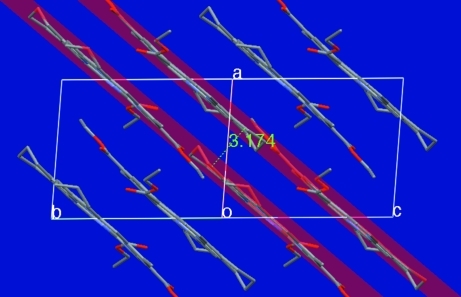
Packing diagram of the title compound.

## supplementary crystallographic information

### Comment

Investigations on the porphyrin isomers such as porphycenes, N-confused porphyrins, corroles, *etc*. have attracted much attention because their unique core geometry often leads to different physicochemical properties both in artificial model complexes and hemeproteins (Erben *et al.*, 2000; Gross *et al.*, 2000; Ohgo *et al.*, 2002; Sessler *et al.*, 2000). The accumulations of the structure analyses on these unique core geometry should be of great advantage in designing the new artificial materials or artificial proteins. It is also quite important to elucidate the electronic and steric effects of the peripheral substituents of the macrocycles, since the reconstitution experiments of these substituted macrocycles instead of normal hemes frequently represent some unusual functional activities. In this paper, we report structure analysis of free-base corrphycene which possess large cyclohexyl rings at pyrrole β-positions. Figure 1 shows the *ORTEP* drawing of the title compound with atom numbering. The corrphycene macrocycle shows nearly planar structure where the r.m.s. deviation of the peripheral 20 carbon atoms and 4 nitrogen atoms is only 0.054 (3) Å. The central N_4_ cavity shows trapezoidal geometry with N1···N2 = 2.578, N2···N3 = 2.774, N3···N4 = 3.538 and N4···N1 = 2.776 Å. Thus, the surface area within the N_4_ coordinating core is 8.358 Å^2^, which is significantly smaller than that of the corresponding free-base porphyrin, 8.503 Å^2^ in OEP (OEP: dianion of 2,3,7,8,12,13,17,18-octaethylporphyrin; Lauher *et al.*, 1973). Two intramolecular hydrogen bonds are found; N1—H1···N4 and N3—H3···N2. The detailed structure analysis clarified that the co-crystallization of the free-base corrphycene together with corrphycenato-Cu(II), which is an intermediate product in the template synthesis, is occurred in the recrystallization process. It is quite interesting that the free-base and it's metal complex are co-crystallized in this manner. This phenomenon should be ascribed to the structural similarity, such as core geometry and planarity, in both compounds. Figure 2 shows a packing diagram, which exhibits the layered structure of the title compound. The distance between the layers is determined to be 3.174 Å.

### Experimental

Ethyl 4,5,6,7-tetrahydro-2*H*-isoindole-1-carboxylate was prepared from 2-formyl cyclohexanone and ethyl glycine hydrochloride according to the reported method (Hombrecher *et al.*, 1992). The compound was derived into 12,17-diethoxycarbonyl-11,18-dimethyl-2,3:6,7-dibutanocorrphycene according to the reported method (Neya *et al.*, 1998). The cyclization of the α,ω-free tetrapyrrole was accomplished by copper(II) chloride. The chelating copper is readily removed by sulfuric acid. NMR spectra of the obtained title compound showed slight broadening probably because of the contamination of the small amount of its metal complex. Futher purification was carried out, however, no changes were observed in the NMR spectra. The solid thus obtained was recrystallized from chloroform solution.

### Refinement

The contamination of a small amount of the corrphycenato-Cu(II) was observed. The ratio of the free-base corrphycene to corrphycenato-Cu(II) complex are determined to be 0.97/0.03 based on the electron density of the copper atom. Since the occupancy factor for the minor conponent, corrphycenato-Cu(II), was too low, it was impossible to separate atomic coordinates and displacement parameters of free-base and metal complex. Hence, bond lengths and angles involving the copper atom listed in the cif could not be accurate. The highest residual electron density peak is located 1.58 Å from atom C22. The positional parameters for H1 and H3 were refined at the beginning of the refinement and  were fixed later to refine the occupancy factors. Other H atoms were refined using a riding model. The positional parameters of H atoms were constrained to have the C—H distances of 0.96 Å for primary, 0.97 Å for secondary, and 0.93 Å for aromatic. Hydrogen *U* values constrained to 1.2 times the equivalent isotropic *U* of the atoms to which they are attached (1.5 for methyl groups).

### Crystal data

**Table:**

[Cu(C_36_H_36_N_4_O_4_)]_0.034_·0.966C_36_H_38_N_4_O_4_	*Z* = 2
*M**_r_* = 592.80	*F*(000) = 630.8
Triclinic, *P*1	*D*_x_ = 1.344 Mg m^−^^3^
Hall symbol: -P 1	Mo *K*α radiation, λ = 0.71073 Å
*a* = 8.8759 (5) Å	Cell parameters from 9696 reflections
*b* = 13.2493 (8) Å	θ = 3.0–27.5°
*c* = 13.2891 (7) Å	µ = 0.11 mm^−^^1^
α = 108.496 (2)°	*T* = 296 K
β = 90.708 (2)°	Block, purple
γ = 98.142 (2)°	0.31 × 0.25 × 0.10 mm
*V* = 1464.4 (1) Å^3^	

### Data collection

**Table:**

Rigaku RAPID diffractometer	6677 independent reflections
Radiation source: fine-focus sealed tube	3707 reflections with *I* > 2σ(*I*)
graphite	*R*_int_ = 0.044
Detector resolution: 10 pixels mm^-1^	θ_max_ = 27.5°, θ_min_ = 3.0°
ω–scan	*h* = −11→11
Absorption correction: multi-scan (*ABSCOR*; Higashi, 1995)	*k* = −17→17
*T*_min_ = 0.788, *T*_max_ = 0.923	*l* = −17→17
14734 measured reflections	

### Refinement

**Table:**

Refinement on *F*^2^	Primary atom site location: structure-invariant direct methods
Least-squares matrix: full	Secondary atom site location: difference Fourier map
*R*[*F*^2^ > 2σ(*F*^2^)] = 0.072	Hydrogen site location: inferred from neighbouring sites
*wR*(*F*^2^) = 0.246	H-atom parameters constrained
*S* = 1.15	*w* = 1/[σ^2^(*F*_o_^2^) + (0.1301*P*)^2^] where *P* = (*F*_o_^2^ + 2*F*_c_^2^)/3
6677 reflections	(Δ/σ)_max_ < 0.001
412 parameters	Δρ_max_ = 1.69 e Å^−^^3^
0 restraints	Δρ_min_ = −0.58 e Å^−^^3^

### Special details

**Table:**

Geometry. All e.s.d.'s (except the e.s.d. in the dihedral angle between two l.s. planes) are estimated using the full covariance matrix. The cell e.s.d.'s are taken into account individually in the estimation of e.s.d.'s in distances, angles and torsion angles; correlations between e.s.d.'s in cell parameters are only used when they are defined by crystal symmetry. An approximate (isotropic) treatment of cell e.s.d.'s is used for estimating e.s.d.'s involving l.s. planes.
Refinement. Refinement of *F*^2^ against ALL reflections. The weighted *R*-factor *wR* and goodness of fit *S* are based on *F*^2^, conventional *R*-factors *R* are based on *F*, with *F* set to zero for negative *F*^2^. The threshold expression of *F*^2^ > σ(*F*^2^) is used only for calculating *R*-factors(gt) *etc*. and is not relevant to the choice of reflections for refinement. *R*-factors based on *F*^2^ are statistically about twice as large as those based on *F*, and *R*- factors based on ALL data will be even larger.

### Fractional atomic coordinates and isotropic or equivalent isotropic displacement parameters (Å^2^)

**Table:**

	*x*	*y*	*z*	*U*_iso_*/*U*_eq_	Occ. (<1)
Cu	0.0199 (11)	−0.0170 (8)	0.6898 (8)	0.023 (4)*	0.0342 (17)
H1	−0.0579	−0.0371	0.7551	0.066 (14)*	0.9658 (17)
H3	0.1166	0.0014	0.6183	0.090 (18)*	0.9658 (17)
O1	0.5049 (3)	0.24582 (18)	0.5059 (2)	0.0473 (6)	
O2	0.5345 (2)	0.10428 (16)	0.36605 (17)	0.0342 (5)	
O3	0.2084 (4)	0.46877 (19)	0.9008 (2)	0.0608 (8)	
O4	0.2074 (3)	0.45953 (18)	1.06523 (19)	0.0498 (7)	
N1	−0.1223 (3)	−0.0788 (2)	0.7810 (2)	0.0307 (6)	
N2	−0.0363 (3)	−0.16368 (19)	0.5921 (2)	0.0301 (6)	
N3	0.1672 (3)	0.00372 (19)	0.5646 (2)	0.0285 (5)	
N4	0.0455 (3)	0.12471 (19)	0.82171 (19)	0.0294 (6)	
C1	−0.1757 (3)	−0.0399 (2)	0.8790 (2)	0.0300 (6)	
C2	−0.2824 (3)	−0.1250 (2)	0.8933 (2)	0.0309 (7)	
C3	−0.2908 (3)	−0.2135 (2)	0.8016 (2)	0.0288 (6)	
C4	−0.1868 (3)	−0.1822 (2)	0.7303 (2)	0.0293 (6)	
C5	−0.1381 (3)	−0.2301 (2)	0.6262 (2)	0.0278 (6)	
C6	−0.1705 (3)	−0.3366 (2)	0.5470 (2)	0.0299 (6)	
C7	−0.0859 (4)	−0.3315 (2)	0.4628 (2)	0.0316 (7)	
C8	−0.0022 (3)	−0.2228 (2)	0.4921 (2)	0.0295 (6)	
C9	0.0992 (3)	−0.1786 (2)	0.4340 (2)	0.0306 (7)	
H9	0.1155	−0.2226	0.3663	0.037*	
C10	0.1798 (3)	−0.0737 (2)	0.4675 (2)	0.0301 (6)	
C11	0.2930 (3)	−0.0293 (2)	0.4121 (2)	0.0295 (6)	
C12	0.3471 (3)	0.0748 (2)	0.4781 (2)	0.0291 (6)	
C13	0.2670 (3)	0.0956 (2)	0.5738 (2)	0.0276 (6)	
C14	0.2903 (3)	0.1949 (2)	0.6577 (2)	0.0311 (7)	
H14	0.3664	0.2425	0.6418	0.037*	
C15	0.2366 (3)	0.2440 (2)	0.7553 (2)	0.0312 (7)	
H15	0.2841	0.3151	0.7830	0.037*	
C16	0.1310 (3)	0.2192 (2)	0.8265 (2)	0.0300 (6)	
C17	0.1087 (3)	0.3021 (2)	0.9271 (2)	0.0310 (7)	
C18	0.0115 (3)	0.2540 (2)	0.9838 (2)	0.0300 (6)	
C19	−0.0280 (3)	0.1427 (2)	0.9157 (2)	0.0299 (6)	
C20	−0.1292 (3)	0.0661 (2)	0.9425 (2)	0.0301 (6)	
H20	−0.1691	0.0880	1.0090	0.036*	
C21	−0.3712 (4)	−0.1235 (3)	0.9887 (3)	0.0351 (7)	
H21A	−0.4455	−0.0750	0.9958	0.042*	
H21B	−0.3024	−0.0962	1.0519	0.042*	
C22	−0.4532 (4)	−0.2350 (3)	0.9811 (3)	0.0443 (8)	
H22A	−0.3830	−0.2734	1.0054	0.053*	
H22B	−0.5367	−0.2272	1.0280	0.053*	
C23	−0.5151 (4)	−0.3006 (3)	0.8697 (3)	0.0419 (8)	
H23A	−0.5915	−0.2650	0.8478	0.050*	
H23B	−0.5650	−0.3705	0.8701	0.050*	
C24	−0.3943 (4)	−0.3170 (2)	0.7888 (3)	0.0348 (7)	
H24A	−0.3347	−0.3699	0.7978	0.042*	
H24B	−0.4431	−0.3445	0.7177	0.042*	
C25	−0.2743 (4)	−0.4357 (2)	0.5472 (3)	0.0379 (7)	
H25A	−0.3795	−0.4250	0.5411	0.045*	
H25B	−0.2580	−0.4490	0.6139	0.045*	
C26	−0.2449 (4)	−0.5331 (3)	0.4549 (3)	0.0474 (9)	
H26A	−0.1542	−0.5583	0.4730	0.057*	
H26B	−0.3301	−0.5908	0.4444	0.057*	
C27	−0.2240 (5)	−0.5075 (3)	0.3518 (3)	0.0485 (9)	
H27A	−0.2122	−0.5727	0.2950	0.058*	
H27B	−0.3142	−0.4817	0.3336	0.058*	
C28	−0.0856 (4)	−0.4230 (2)	0.3618 (3)	0.0354 (7)	
H28A	0.0065	−0.4547	0.3616	0.042*	
H28B	−0.0869	−0.3966	0.3016	0.042*	
C29	0.3370 (4)	−0.0890 (3)	0.3028 (2)	0.0345 (7)	
H29A	0.3439	−0.0425	0.2600	0.052*	
H29B	0.2612	−0.1509	0.2707	0.052*	
H29C	0.4339	−0.1118	0.3080	0.052*	
C30	0.4677 (3)	0.1519 (2)	0.4546 (2)	0.0303 (6)	
C31	0.6546 (4)	0.1722 (2)	0.3344 (2)	0.0335 (7)	
H31A	0.7376	0.1991	0.3888	0.040*	
H31B	0.6159	0.2331	0.3235	0.040*	
C32	0.7086 (4)	0.1028 (3)	0.2324 (3)	0.0411 (8)	
H32A	0.7390	0.0401	0.2431	0.062*	
H32B	0.7938	0.1429	0.2111	0.062*	
H32C	0.6274	0.0813	0.1780	0.062*	
C33	0.1799 (4)	0.4170 (2)	0.9603 (3)	0.0366 (7)	
C34	0.2742 (6)	0.5728 (3)	1.1055 (3)	0.0632 (12)	
H34A	0.3510	0.5880	1.0588	0.076*	
H34B	0.1958	0.6171	1.1067	0.076*	
C35	0.3420 (6)	0.5982 (3)	1.2102 (4)	0.0796 (16)	
H35A	0.2674	0.5788	1.2552	0.119*	
H35B	0.3787	0.6741	1.2381	0.119*	
H35C	0.4256	0.5589	1.2079	0.119*	
C36	−0.0458 (4)	0.3008 (2)	1.0907 (3)	0.0354 (7)	
H36A	0.0165	0.2874	1.1432	0.053*	
H36B	−0.1493	0.2683	1.0917	0.053*	
H36C	−0.0416	0.3771	1.1063	0.053*	

### Atomic displacement parameters (Å^2^)

**Table:**

	*U*^11^	*U*^22^	*U*^33^	*U*^12^	*U*^13^	*U*^23^
O1	0.0499 (14)	0.0317 (12)	0.0501 (15)	−0.0069 (11)	0.0205 (12)	0.0036 (11)
O2	0.0380 (12)	0.0289 (11)	0.0339 (12)	−0.0020 (9)	0.0101 (9)	0.0104 (9)
O3	0.109 (2)	0.0311 (13)	0.0394 (15)	−0.0042 (14)	0.0083 (15)	0.0134 (11)
O4	0.0808 (19)	0.0276 (11)	0.0339 (14)	−0.0099 (12)	−0.0029 (12)	0.0081 (10)
N1	0.0306 (13)	0.0276 (12)	0.0311 (14)	−0.0015 (11)	0.0012 (11)	0.0081 (11)
N2	0.0295 (13)	0.0264 (12)	0.0327 (14)	−0.0010 (11)	0.0008 (10)	0.0095 (10)
N3	0.0313 (13)	0.0253 (12)	0.0275 (13)	0.0016 (11)	0.0036 (10)	0.0077 (10)
N4	0.0296 (13)	0.0288 (13)	0.0291 (14)	0.0026 (11)	0.0027 (10)	0.0090 (10)
C1	0.0307 (15)	0.0285 (15)	0.0310 (16)	0.0009 (13)	0.0029 (12)	0.0116 (12)
C2	0.0319 (15)	0.0304 (15)	0.0301 (16)	0.0045 (13)	0.0031 (12)	0.0095 (12)
C3	0.0258 (14)	0.0254 (14)	0.0360 (17)	0.0005 (12)	0.0016 (12)	0.0124 (12)
C4	0.0266 (15)	0.0275 (14)	0.0330 (17)	0.0009 (12)	−0.0020 (12)	0.0102 (12)
C5	0.0246 (14)	0.0239 (14)	0.0353 (17)	0.0008 (12)	0.0015 (12)	0.0113 (12)
C6	0.0279 (15)	0.0263 (14)	0.0347 (17)	0.0023 (12)	0.0003 (12)	0.0096 (12)
C7	0.0349 (16)	0.0278 (15)	0.0306 (17)	0.0028 (13)	0.0001 (12)	0.0081 (12)
C8	0.0264 (14)	0.0288 (14)	0.0318 (16)	−0.0001 (12)	0.0027 (12)	0.0096 (12)
C9	0.0327 (16)	0.0288 (15)	0.0273 (16)	0.0013 (13)	−0.0007 (12)	0.0066 (12)
C10	0.0285 (15)	0.0299 (15)	0.0321 (16)	0.0036 (13)	0.0011 (12)	0.0105 (12)
C11	0.0306 (15)	0.0273 (14)	0.0304 (16)	0.0006 (13)	0.0010 (12)	0.0109 (12)
C12	0.0281 (15)	0.0285 (15)	0.0318 (16)	0.0035 (13)	0.0015 (12)	0.0117 (12)
C13	0.0256 (14)	0.0291 (15)	0.0303 (16)	0.0015 (12)	0.0046 (11)	0.0136 (12)
C14	0.0316 (15)	0.0288 (15)	0.0328 (17)	−0.0016 (13)	0.0022 (12)	0.0124 (13)
C15	0.0321 (16)	0.0270 (14)	0.0321 (17)	−0.0025 (13)	0.0002 (12)	0.0090 (12)
C16	0.0321 (16)	0.0253 (14)	0.0319 (16)	−0.0007 (13)	0.0020 (12)	0.0103 (12)
C17	0.0333 (16)	0.0235 (14)	0.0344 (17)	−0.0013 (13)	−0.0011 (13)	0.0094 (12)
C18	0.0304 (15)	0.0304 (15)	0.0287 (16)	0.0003 (13)	0.0015 (12)	0.0106 (12)
C19	0.0299 (15)	0.0308 (15)	0.0288 (16)	0.0032 (13)	0.0016 (12)	0.0098 (12)
C20	0.0307 (15)	0.0278 (15)	0.0301 (16)	0.0003 (13)	0.0025 (12)	0.0087 (12)
C21	0.0390 (17)	0.0330 (16)	0.0344 (17)	0.0068 (14)	0.0044 (13)	0.0119 (13)
C22	0.0430 (19)	0.0433 (19)	0.045 (2)	−0.0026 (16)	0.0066 (16)	0.0165 (16)
C23	0.046 (2)	0.0388 (18)	0.042 (2)	0.0018 (16)	0.0080 (15)	0.0163 (15)
C24	0.0376 (17)	0.0303 (16)	0.0367 (18)	0.0006 (14)	0.0011 (13)	0.0132 (13)
C25	0.0377 (17)	0.0311 (16)	0.0420 (19)	−0.0014 (14)	0.0079 (14)	0.0103 (14)
C26	0.050 (2)	0.0319 (17)	0.050 (2)	−0.0057 (16)	0.0081 (17)	0.0044 (15)
C27	0.056 (2)	0.0370 (18)	0.042 (2)	−0.0061 (17)	0.0051 (17)	0.0036 (15)
C28	0.0391 (17)	0.0273 (15)	0.0368 (18)	0.0016 (13)	0.0012 (13)	0.0078 (13)
C29	0.0354 (16)	0.0348 (16)	0.0324 (17)	0.0022 (14)	0.0048 (13)	0.0109 (13)
C30	0.0288 (15)	0.0292 (15)	0.0323 (17)	−0.0022 (13)	0.0024 (12)	0.0117 (13)
C31	0.0320 (16)	0.0337 (16)	0.0344 (17)	−0.0034 (14)	0.0035 (13)	0.0142 (13)
C32	0.048 (2)	0.0457 (19)	0.0343 (18)	0.0087 (17)	0.0110 (15)	0.0183 (15)
C33	0.0456 (19)	0.0280 (15)	0.0357 (18)	0.0050 (14)	0.0023 (14)	0.0101 (13)
C34	0.111 (4)	0.0269 (17)	0.044 (2)	−0.013 (2)	−0.007 (2)	0.0112 (16)
C35	0.086 (3)	0.034 (2)	0.103 (4)	−0.006 (2)	−0.032 (3)	0.007 (2)
C36	0.0399 (17)	0.0287 (15)	0.0365 (18)	0.0033 (14)	0.0080 (14)	0.0099 (13)

### Geometric parameters (Å, °)

**Table:**

Cu—N2	1.954 (10)	C17—C18	1.373 (4)
Cu—N1	2.026 (10)	C17—C33	1.485 (4)
Cu—N4	2.105 (10)	C18—C19	1.453 (4)
Cu—N3	2.189 (10)	C18—C36	1.487 (4)
O1—C30	1.209 (4)	C19—C20	1.394 (4)
O2—C30	1.337 (4)	C20—H20	0.9300
O2—C31	1.450 (4)	C21—C22	1.523 (5)
O3—C33	1.208 (4)	C21—H21A	0.9700
O4—C33	1.332 (4)	C21—H21B	0.9700
O4—C34	1.456 (4)	C22—C23	1.510 (5)
N1—C4	1.357 (4)	C22—H22A	0.9700
N1—C1	1.358 (4)	C22—H22B	0.9700
N1—H1	0.886	C23—C24	1.516 (5)
N2—C5	1.355 (4)	C23—H23A	0.9700
N2—C8	1.374 (4)	C23—H23B	0.9700
N3—C13	1.371 (4)	C24—H24A	0.9700
N3—C10	1.388 (4)	C24—H24B	0.9700
N3—H3	0.854	C25—C26	1.528 (4)
N4—C16	1.351 (4)	C25—H25A	0.9700
N4—C19	1.385 (4)	C25—H25B	0.9700
C1—C20	1.390 (4)	C26—C27	1.519 (5)
C1—C2	1.429 (4)	C26—H26A	0.9700
C2—C3	1.390 (4)	C26—H26B	0.9700
C2—C21	1.497 (4)	C27—C28	1.515 (5)
C3—C4	1.444 (4)	C27—H27A	0.9700
C3—C24	1.498 (4)	C27—H27B	0.9700
C4—C5	1.427 (4)	C28—H28A	0.9700
C5—C6	1.456 (4)	C28—H28B	0.9700
C6—C7	1.369 (4)	C29—H29A	0.9600
C6—C25	1.494 (4)	C29—H29B	0.9600
C7—C8	1.453 (4)	C29—H29C	0.9600
C7—C28	1.497 (4)	C31—C32	1.504 (4)
C8—C9	1.379 (4)	C31—H31A	0.9700
C9—C10	1.400 (4)	C31—H31B	0.9700
C9—H9	0.9300	C32—H32A	0.9600
C10—C11	1.423 (4)	C32—H32B	0.9600
C11—C12	1.392 (4)	C32—H32C	0.9600
C11—C29	1.503 (4)	C34—C35	1.427 (6)
C12—C13	1.433 (4)	C34—H34A	0.9700
C12—C30	1.479 (4)	C34—H34B	0.9700
C13—C14	1.416 (4)	C35—H35A	0.9600
C14—C15	1.379 (4)	C35—H35B	0.9600
C14—H14	0.9300	C35—H35C	0.9600
C15—C16	1.419 (4)	C36—H36A	0.9600
C15—H15	0.9300	C36—H36B	0.9600
C16—C17	1.473 (4)	C36—H36C	0.9600
			
N2—Cu—N1	80.7 (4)	C22—C21—H21B	109.2
N2—Cu—N4	165.0 (6)	H21A—C21—H21B	107.9
N1—Cu—N4	84.3 (4)	C23—C22—C21	112.9 (3)
N2—Cu—N3	83.9 (4)	C23—C22—H22A	109.0
N1—Cu—N3	164.4 (5)	C21—C22—H22A	109.0
N4—Cu—N3	110.9 (4)	C23—C22—H22B	109.0
C30—O2—C31	116.0 (2)	C21—C22—H22B	109.0
C33—O4—C34	116.5 (3)	H22A—C22—H22B	107.8
C4—N1—C1	111.7 (3)	C22—C23—C24	113.7 (3)
C4—N1—Cu	112.8 (3)	C22—C23—H23A	108.8
C1—N1—Cu	135.4 (3)	C24—C23—H23A	108.8
C4—N1—H1	127.1	C22—C23—H23B	108.8
C5—N2—C8	106.3 (2)	C24—C23—H23B	108.8
C5—N2—Cu	116.1 (3)	H23A—C23—H23B	107.7
C8—N2—Cu	137.5 (4)	C3—C24—C23	111.0 (3)
C13—N3—C10	109.8 (2)	C3—C24—H24A	109.4
C13—N3—Cu	124.0 (3)	C23—C24—H24A	109.4
C10—N3—Cu	126.1 (3)	C3—C24—H24B	109.4
C13—N3—H3	117.9	C23—C24—H24B	109.4
C10—N3—H3	132.0	H24A—C24—H24B	108.0
C16—N4—C19	106.7 (2)	C6—C25—C26	110.7 (3)
C16—N4—Cu	125.3 (3)	C6—C25—H25A	109.5
C19—N4—Cu	127.9 (3)	C26—C25—H25A	109.5
N1—C1—C20	121.0 (3)	C6—C25—H25B	109.5
N1—C1—C2	106.8 (3)	C26—C25—H25B	109.5
C20—C1—C2	132.2 (3)	H25A—C25—H25B	108.1
C3—C2—C1	107.9 (3)	C27—C26—C25	112.6 (3)
C3—C2—C21	124.3 (3)	C27—C26—H26A	109.1
C1—C2—C21	127.8 (3)	C25—C26—H26A	109.1
C2—C3—C4	106.8 (3)	C27—C26—H26B	109.1
C2—C3—C24	122.2 (3)	C25—C26—H26B	109.1
C4—C3—C24	131.0 (3)	H26A—C26—H26B	107.8
N1—C4—C5	115.8 (3)	C28—C27—C26	111.2 (3)
N1—C4—C3	106.8 (3)	C28—C27—H27A	109.4
C5—C4—C3	137.4 (3)	C26—C27—H27A	109.4
N2—C5—C4	114.4 (3)	C28—C27—H27B	109.4
N2—C5—C6	111.0 (3)	C26—C27—H27B	109.4
C4—C5—C6	134.5 (3)	H27A—C27—H27B	108.0
C7—C6—C5	105.9 (3)	C7—C28—C27	109.9 (3)
C7—C6—C25	123.0 (3)	C7—C28—H28A	109.7
C5—C6—C25	131.1 (3)	C27—C28—H28A	109.7
C6—C7—C8	106.6 (3)	C7—C28—H28B	109.7
C6—C7—C28	124.5 (3)	C27—C28—H28B	109.7
C8—C7—C28	128.8 (3)	H28A—C28—H28B	108.2
N2—C8—C9	121.3 (3)	C11—C29—H29A	109.5
N2—C8—C7	110.1 (3)	C11—C29—H29B	109.5
C9—C8—C7	128.6 (3)	H29A—C29—H29B	109.5
C8—C9—C10	126.1 (3)	C11—C29—H29C	109.5
C8—C9—H9	117.0	H29A—C29—H29C	109.5
C10—C9—H9	117.0	H29B—C29—H29C	109.5
N3—C10—C9	125.0 (3)	O1—C30—O2	122.4 (3)
N3—C10—C11	108.1 (3)	O1—C30—C12	126.7 (3)
C9—C10—C11	126.9 (3)	O2—C30—C12	110.9 (2)
C12—C11—C10	106.5 (3)	O2—C31—C32	106.4 (3)
C12—C11—C29	129.9 (3)	O2—C31—H31A	110.4
C10—C11—C29	123.6 (3)	C32—C31—H31A	110.4
C11—C12—C13	108.8 (3)	O2—C31—H31B	110.4
C11—C12—C30	126.1 (3)	C32—C31—H31B	110.4
C13—C12—C30	125.1 (3)	H31A—C31—H31B	108.6
N3—C13—C14	129.2 (3)	C31—C32—H32A	109.5
N3—C13—C12	106.8 (2)	C31—C32—H32B	109.5
C14—C13—C12	124.0 (3)	H32A—C32—H32B	109.5
C15—C14—C13	140.4 (3)	C31—C32—H32C	109.5
C15—C14—H14	109.8	H32A—C32—H32C	109.5
C13—C14—H14	109.8	H32B—C32—H32C	109.5
C14—C15—C16	139.4 (3)	O3—C33—O4	122.4 (3)
C14—C15—H15	110.3	O3—C33—C17	125.0 (3)
C16—C15—H15	110.3	O4—C33—C17	112.6 (3)
N4—C16—C15	130.2 (3)	C35—C34—O4	110.2 (3)
N4—C16—C17	109.5 (3)	C35—C34—H34A	109.6
C15—C16—C17	120.2 (3)	O4—C34—H34A	109.6
C18—C17—C16	107.7 (3)	C35—C34—H34B	109.6
C18—C17—C33	126.6 (3)	O4—C34—H34B	109.6
C16—C17—C33	125.6 (3)	H34A—C34—H34B	108.1
C17—C18—C19	104.9 (3)	C34—C35—H35A	109.5
C17—C18—C36	129.6 (3)	C34—C35—H35B	109.5
C19—C18—C36	125.5 (3)	H35A—C35—H35B	109.5
N4—C19—C20	125.4 (3)	C34—C35—H35C	109.5
N4—C19—C18	111.2 (3)	H35A—C35—H35C	109.5
C20—C19—C18	123.5 (3)	H35B—C35—H35C	109.5
C1—C20—C19	125.9 (3)	C18—C36—H36A	109.5
C1—C20—H20	117.0	C18—C36—H36B	109.5
C19—C20—H20	117.0	H36A—C36—H36B	109.5
C2—C21—C22	112.1 (3)	C18—C36—H36C	109.5
C2—C21—H21A	109.2	H36A—C36—H36C	109.5
C22—C21—H21A	109.2	H36B—C36—H36C	109.5
C2—C21—H21B	109.2		
			
N2—Cu—N1—C4	−2.9 (4)	C8—C9—C10—C11	175.0 (3)
N4—Cu—N1—C4	178.1 (2)	N3—C10—C11—C12	0.4 (3)
N3—Cu—N1—C4	−13.5 (19)	C9—C10—C11—C12	−176.7 (3)
N2—Cu—N1—C1	−178.4 (3)	N3—C10—C11—C29	−179.1 (3)
N4—Cu—N1—C1	2.6 (5)	C9—C10—C11—C29	3.8 (5)
N3—Cu—N1—C1	171.0 (16)	C10—C11—C12—C13	−0.6 (3)
N1—Cu—N2—C5	2.1 (4)	C29—C11—C12—C13	178.8 (3)
N4—Cu—N2—C5	6(2)	C10—C11—C12—C30	179.2 (3)
N3—Cu—N2—C5	179.3 (2)	C29—C11—C12—C30	−1.4 (5)
N1—Cu—N2—C8	−178.9 (3)	C10—N3—C13—C14	178.4 (3)
N4—Cu—N2—C8	−175.1 (17)	Cu—N3—C13—C14	−4.3 (5)
N3—Cu—N2—C8	−1.8 (6)	C10—N3—C13—C12	−0.4 (3)
N2—Cu—N3—C13	−175.6 (2)	Cu—N3—C13—C12	176.9 (3)
N1—Cu—N3—C13	−165.1 (17)	C11—C12—C13—N3	0.7 (3)
N4—Cu—N3—C13	2.6 (5)	C30—C12—C13—N3	−179.2 (2)
N2—Cu—N3—C10	1.2 (5)	C11—C12—C13—C14	−178.3 (3)
N1—Cu—N3—C10	12 (2)	C30—C12—C13—C14	1.9 (4)
N4—Cu—N3—C10	179.4 (3)	N3—C13—C14—C15	−0.4 (6)
N2—Cu—N4—C16	178.0 (18)	C12—C13—C14—C15	178.2 (3)
N1—Cu—N4—C16	−178.2 (2)	C13—C14—C15—C16	2.9 (7)
N3—Cu—N4—C16	5.1 (6)	C19—N4—C16—C15	175.0 (3)
N2—Cu—N4—C19	−7(2)	Cu—N4—C16—C15	−9.2 (5)
N1—Cu—N4—C19	−3.3 (5)	C19—N4—C16—C17	−1.4 (3)
N3—Cu—N4—C19	−180.0 (3)	Cu—N4—C16—C17	174.4 (3)
C4—N1—C1—C20	−177.3 (3)	C14—C15—C16—N4	4.7 (6)
Cu—N1—C1—C20	−1.7 (6)	C14—C15—C16—C17	−179.2 (3)
C4—N1—C1—C2	0.5 (3)	N4—C16—C17—C18	2.0 (3)
Cu—N1—C1—C2	176.0 (4)	C15—C16—C17—C18	−174.8 (3)
N1—C1—C2—C3	−0.5 (3)	N4—C16—C17—C33	−178.3 (3)
C20—C1—C2—C3	176.8 (3)	C15—C16—C17—C33	4.9 (4)
N1—C1—C2—C21	179.1 (3)	C16—C17—C18—C19	−1.7 (3)
C20—C1—C2—C21	−3.6 (5)	C33—C17—C18—C19	178.6 (3)
C1—C2—C3—C4	0.4 (3)	C16—C17—C18—C36	178.8 (3)
C21—C2—C3—C4	−179.2 (3)	C33—C17—C18—C36	−1.0 (5)
C1—C2—C3—C24	−179.1 (2)	C16—N4—C19—C20	179.2 (3)
C21—C2—C3—C24	1.3 (4)	Cu—N4—C19—C20	3.6 (5)
C1—N1—C4—C5	179.8 (2)	C16—N4—C19—C18	0.3 (3)
Cu—N1—C4—C5	3.2 (4)	Cu—N4—C19—C18	−175.3 (4)
C1—N1—C4—C3	−0.2 (3)	C17—C18—C19—N4	0.9 (3)
Cu—N1—C4—C3	−176.8 (3)	C36—C18—C19—N4	−179.5 (3)
C2—C3—C4—N1	−0.1 (3)	C17—C18—C19—C20	−178.0 (3)
C24—C3—C4—N1	179.3 (3)	C36—C18—C19—C20	1.6 (5)
C2—C3—C4—C5	179.9 (3)	N1—C1—C20—C19	0.4 (4)
C24—C3—C4—C5	−0.6 (5)	C2—C1—C20—C19	−176.7 (3)
C8—N2—C5—C4	179.7 (2)	N4—C19—C20—C1	−1.5 (5)
Cu—N2—C5—C4	−1.1 (4)	C18—C19—C20—C1	177.3 (3)
C8—N2—C5—C6	0.9 (3)	C3—C2—C21—C22	8.9 (4)
Cu—N2—C5—C6	−179.9 (3)	C1—C2—C21—C22	−170.6 (3)
N1—C4—C5—N2	−1.5 (4)	C2—C21—C22—C23	−37.3 (4)
C3—C4—C5—N2	178.5 (3)	C21—C22—C23—C24	58.1 (4)
N1—C4—C5—C6	177.0 (3)	C2—C3—C24—C23	16.9 (4)
C3—C4—C5—C6	−3.0 (6)	C4—C3—C24—C23	−162.5 (3)
N2—C5—C6—C7	−1.0 (3)	C22—C23—C24—C3	−45.9 (4)
C4—C5—C6—C7	−179.5 (3)	C7—C6—C25—C26	12.8 (4)
N2—C5—C6—C25	−179.5 (3)	C5—C6—C25—C26	−168.9 (3)
C4—C5—C6—C25	2.0 (5)	C6—C25—C26—C27	−43.0 (4)
C5—C6—C7—C8	0.6 (3)	C25—C26—C27—C28	62.6 (4)
C25—C6—C7—C8	179.3 (3)	C6—C7—C28—C27	17.6 (4)
C5—C6—C7—C28	−179.0 (3)	C8—C7—C28—C27	−162.0 (3)
C25—C6—C7—C28	−0.4 (5)	C26—C27—C28—C7	−46.9 (4)
C5—N2—C8—C9	179.9 (3)	C31—O2—C30—O1	−0.2 (4)
Cu—N2—C8—C9	1.0 (6)	C31—O2—C30—C12	179.7 (2)
C5—N2—C8—C7	−0.5 (3)	C11—C12—C30—O1	171.8 (3)
Cu—N2—C8—C7	−179.4 (4)	C13—C12—C30—O1	−8.4 (5)
C6—C7—C8—N2	−0.1 (3)	C11—C12—C30—O2	−8.2 (4)
C28—C7—C8—N2	179.5 (3)	C13—C12—C30—O2	171.6 (2)
C6—C7—C8—C9	179.4 (3)	C30—O2—C31—C32	−178.4 (2)
C28—C7—C8—C9	−1.0 (5)	C34—O4—C33—O3	0.1 (5)
N2—C8—C9—C10	1.3 (5)	C34—O4—C33—C17	−178.7 (3)
C7—C8—C9—C10	−178.3 (3)	C18—C17—C33—O3	−146.8 (4)
C13—N3—C10—C9	177.2 (3)	C16—C17—C33—O3	33.5 (5)
Cu—N3—C10—C9	0.0 (5)	C18—C17—C33—O4	31.9 (4)
C13—N3—C10—C11	0.0 (3)	C16—C17—C33—O4	−147.8 (3)
Cu—N3—C10—C11	−177.2 (3)	C33—O4—C34—C35	−161.3 (4)
C8—C9—C10—N3	−1.6 (5)		

### Hydrogen-bond geometry (Å, °)

**Table:**

*D*—H···*A*	*D*—H	H···*A*	*D*···*A*	*D*—H···*A*
N3—H3···N2	0.85	2.33	2.777 (4)	113
N1—H1···N4	0.89	2.11	2.774 (4)	131
